# Microcurrent Reverses Cigarette Smoke-Induced Angiogenesis Impairment in Human Keratinocytes In Vitro

**DOI:** 10.3390/bioengineering9090445

**Published:** 2022-09-06

**Authors:** Chao Lu, Cosima Prahm, Yangmengfan Chen, Sabrina Ehnert, Helen Rinderknecht, Colin D. McCaig, Andreas K. Nussler, Jonas Kolbenschlag

**Affiliations:** 1 Department of Hand-, Plastic, Reconstructive and Burn Surgery, BG Klinik Tuebingen, University of Tuebingen, Schnarrenbergstrasse 95, D-72076 Tuebingen, Germany; 2 Siegfried-Weller Institute for Trauma Research, BG Kinik Tuebingen, University of Tuebingen, Schnarrenbergstrasse 95, D-72070 Tuebingen, Germany; 3 Institute of Medical Sciences, School of Medicine, Medical Sciences and Nutrition, University of Aberdeen, Aberdeen AB25 2ZD, UK

**Keywords:** microcurrent (MC), HaCaT cells, wound healing, cigarette smoke extract, PI3K-Akt, angiogenesis

## Abstract

Cigarette smoking (CS) leads to several adverse health effects, including diseases, disabilities, and even death. Post-operative and trauma patients who smoke have an increased risk for complications, such as delayed bone or wound healing. In clinical trials, microcurrent (MC) has been shown to be a safe, non-invasive, and effective way to accelerate wound healing. Our study aimed to investigate if MC with the strength of 100 μA may be beneficial in treating CS-related healing impairment, especially in regard to angiogenesis. In this study, we investigated the effect of human keratinocyte cells (HaCaT) on angiogenesis after 72 h of cigarette smoke extract (CSE) exposure in the presence or absence of 100 μA MC. Cell viability and proliferation were evaluated by resazurin conversion, Sulforhodamine B, and Calcein-AM/Hoechst 33342 staining; the pro-angiogenic potential of HaCaT cells was evaluated by tube formation assay and angiogenesis array assay; signaling pathway alterations were investigated using Western blot. Constant exposure for 72 h to a 100 μA MC enhanced the angiogenic ability of HaCaT cells, which was mediated through the PI3K-Akt signaling pathway. In conclusion, the current data indicate that 100 μA MC may support wound healing in smoking patients by enhancing angiogenesis.

## 1. Introduction

Tobacco smoking causes several adverse health effects, including many diseases, disabilities, and even death [1]. In Germany, every four minutes, one person dies from a smoking-related illness. In addition, the public health care system faces an annual burden of approximately EUR 97 billion due to tobacco-attributable diseases [2]. In addition to the fact that cigarettes can cause lung cancer, cardiovascular disease, and stroke [3], post-operative and trauma patients who smoke have an increased risk for complications, including delayed wound or bone healing [4]. As a critical process in wound healing, angiogenesis drives the delivery of inflammatory cells and fibroblasts to the wound site [5], thereby providing cytokines, oxygen, nutrients, and removing metabolic waste [6]. Angiogenesis typically occurs around three days (72 h) after injury [7], which means that angiogenesis can be influenced by pathological or therapeutic factors in the early stages of trauma. Current research suggests that the angiogenesis process may be impeded by smoking products, resulting in a compromised blood supply to tissues and a delay in wound healing [8]. Cigarette smoke (CS) inhalation reduced levels of angiogenic markers in rat wound healing [9], which was further substantiated by our lab by showing that smoking adversely affects early vascularization (in newly formed tissue) using an in vitro hematoma model [10].

Treatments to accelerate wound healing and cure chronic wounds are actively sought, and one non-invasive and convenient treatment, electrical stimulation (ES), has been used successfully in research for clinical applications [11]. Based on the existence of endogenous electrical fields at human wound sites (created by the transepithelial potential difference (TEP)) [12], researchers have shown that electrical signals are crucial initiators and regulators of wound cell proliferation [13], migration [14], secretion function [15] and, therefore, act to accelerate wound closure [16]. Microcurrent (MC) is “an electrotherapeutic modality that uses low levels of electrical current (less than 1 mA) to facilitate circulation and cellular healing or to reduce pain or edema” [17]; it is an electrotherapy strategy involving microampere range current [18]. In contrast to other ES methods that can bypass cells, this form of MC can exert effects directly on cells [19] and may even be more effective in stimulating cell physiology and growth [20]. A recent in vitro study by Bravo et al. [21] demonstrated that MC plays a crucial role in wound healing by increasing fibroblast cell proliferation, modulating the inflammatory response, and aiding tissue regeneration compared to controls. In addition, a novel “Microcurrent Dressing” that uses MC on moist wound surfaces to accelerate wound healing has been investigated and the clinical effectiveness has been verified [22]. Microcurrent at 100 μA has been extensively tested and shown to have a clear therapeutic effect on accelerating healing in animal studies [23,24,25], although the exact mechanism behind this is unclear.

Regardless of some positive results from recent clinical trials, the underlying mechanisms of MC on wound healing remain unclear. Animal studies have shown that MC acted as an anti-inflammatory [26] and enhanced blood circulation [27] and re-epithelialization [28]. In vitro experiments can provide vital information; however, the transmission of MC to a cell culture system is challenging [29]. We recently established an experimental ES system to distribute electrical signals to cells in vitro while maintaining a steady cell culture environment [15]. The novel experimental system enhances reproducibility by increasing the number of parallel experimental set-ups and provides an efficient, reliable, and reproducible method for data collection. In our previous study, we explored the effect of electric field signals on the healing-related ability of keratinocytes using the new system. We showed that the electric field enhanced keratinocyte migration, proliferation, cytokine secretion, and that these biological effects were mediated due to an activation of the MAPK signaling pathways [15].

In the present study, we explore the impairment of angiogenesis-related functions of keratinocytes cells induced by CS exposure and the role of MC in counteracting these detrimental effects. In addition, we determine the underlying mechanisms by which MC enhances CS-suppressed angiogenesis in vitro.

## 2. Materials and Methods

### 2.1. Reagents and Cell Lines

Unless otherwise specified, chemical and cell culture medium supplements were obtained from Sigma-Aldrich (Darmstadt, Germany).

The human keratinocyte cell lines (HaCaT cells, obtained from the technology transfer of the German Cancer Research Center (DKFZ), Heidelberg, Germany) were cultured in Gibco Dulbecco’s Modified Eagle Medium with 5% fetal bovine serum (FBS) in an atmosphere of 5% CO_2_ at 37 °C. The medium was replaced every 2 to 3 days. For this study, cells were used in passages 4 to 15.

### 2.2. Preparation of Cigarette Smoke Extract (CSE)

Fresh CSE solution was prepared for each experiment as described by Aspera-Werz et al. [30]. In brief, a lit commercial cigarette (Marlboro, Philip Morris, New York, NY, USA) was attached to a standard gas wash bottle containing serum-free DMEM medium, and the smoke was driven by a peristaltic pump and continuously bubbled through the medium. An aluminum foil was used to protect the CSE solution from light. The optical density at λ = 320 nm (OD_320nm_) serves as the metric to measure CSE concentration; CSE is considered 100% volumetric when OD_320nm_ is 0.7.

After filtration, the CSE solution was further diluted to 1%, 3%, and 5% with HaCaT cells medium (DMEM medium, 5% FCS, 1% penicillin-streptomycin) for the subsequent experiments. CSE exposure of 5% corresponds to approximately smoking 10 cigarettes/day [31].

### 2.3. Resazurin Conversion Assay 

Resazurin conversion assay was used to assess cell viability. In brief, HaCaT cells were treated with 0.025% (*w*/*v*) resazurin solution (in DPBS) to measure mitochondrial activity. Incubation of HaCaT cells with resazurin solution was carried out at 37 °C for 40 min. The intensity of fluorescence was measured at OD_540/590nm_ with a microplate reader (Omega, BMG Labtech, Ortenberg, Germany) and the background fluorescence intensity (well without cells) was subtracted [32,33].

### 2.4. Sulforhodamine B (SRB) Staining Assay

The total protein content of cells was determined by SRB staining assay as reported [34]. HaCaT cells were first fixed with 90% ethanol overnight at −20 °C, then stained with SRB (0.4% *w*/*v* in 1% *v*/*v* acetic acid) for 20 min room temperature. Unbound dye was washed with 1% acetic acid via four times washing, while bound SRB was resolved with TRIS solution (10 mM, unbuffered, pH = 10.5). The final absorbance of the solution was quantified photometrically by a plate-reader (Omega, BMG Labtech, Ortenberg, Germany) at OD_565nm_.

### 2.5. Cell Viability Was Assessed by a Double-Staining Assay Using Calcium-AM/Hoechst 33342

Living cells were visualized by calcein-AM (green fluorescence). Nuclei were labeled by Hoechst 33342 (blue fluorescence). Briefly, after 72 h of CSE administration (1%, 3%, 5% *v*/*v* HaCaT cell culture medium), incubation was performed with Calcein-AM (2 mM) and Hoechst 33342 (1 mg/mL) after three washes with DPBS. The samples were shaken for approximately 30 min, protected from light. Afterward, epifluorescence microscope (EVOS FL, Life Technologies, Darmstadt, Germany) was used to capture the images [35].

### 2.6. MC Application 

The setup to transmit the MC signal was as described previously [15]. In short, a custom-made 6-well plate containing four identical electrotactic chambers was used to build the setup. A continuous MC signal with an intensity of 400 µA is emitted from the electrical signal generator (Vanquish Innovation, New York, NY, USA) and the current was distributed uniformly to each chamber through the electrodes and agar salt bridges. In other words, each chamber received an electrical stimulus of 100 μA intensity.

During the entire culture process, HaCaT cells were exposed to 100 μA MC for 72 h. We have previously established that the pH value and temperature of the cell culture area can be stabilized for at least 72 h with the help of the unique structure of the electrotactic chambers [15].

### 2.7. HaCaT Cells Stimulation and Tube Formation Assay

#### 2.7.1. HaCaT Cells Stimulation and Supernatant Collection

HaCaT cells were treated with either 3% CSE, 100 μA MC, a combination of both, or left untreated (control) for 72 h. To exclude the interference of the CSE, the standard HaCaT medium was replaced and cells were incubated for another 24 h. Afterward, the culture supernatant was collected.

#### 2.7.2. Tube Formation Assay

The effect of HaCaT cells on angiogenesis after 72 h of exposure to 3% CSE in the presence or absence of 100 μA MC was determined by tube formation assay. The assay was performed according to Arnaoutova et al. [36]. In brief, human umbilical vein endothelial cells (HUVECs, obtained from PromoCell (Heidelberg, Germany)) were used as an indicator of the tube formation ability of the supernatant from different groups. Then, 7 μL of gel matrix (ibidi, Gräfelfing, Germany) was uniformly coated on a pre-chilled 24-well plate on ice, then transferred to a 37 °C thermostat for 30 min of polymerization. Next, HUVECs were seeded into each pre-prepared well with a density of 60 × 10^3^ cells/well in 500 μL plain endothelial basal medium-2 (R&D, Minneapolis, MN, USA). Afterward, 500 μL supernatant collected from different groups was added to HUVEC cells in triplicate wells.

After incubation for 16 h, calcein-AM staining was performed (see Section 2.5), and the capillary tube structure was captured via an epifluorescence microscope (EVOS FL, Life Technologies, Darmstadt, Germany). Images were evaluated using ImageJ software throughout (Version 1.8.0_172, NIH, Bethesda, MD, USA). Nb.nodes (nodes are pixels with three neighbors) and Nb.master junctions (master junctions are junctions linking at least three master segments), two critical indicators of tube formation capability [37], were used as the basis for image analysis

### 2.8. Angiogenesis Array Assay

The RayBio Human Growth Factor Array (RayBiotech, Norcross, GA, USA) was used to investigate the expression of angiogenesis-related factors secreted by HaCaT cells under different experimental conditions. Experiments were performed following the manufacturer’s protocol, and the supernatant sample preparation was the same as previously described (see Section 2.7.1). 

Briefly, membranes were washed with washing buffer (provided in the kit) and incubated in blocking buffer (provided in the kit) at RT for 2 h. Next, the membranes were incubated with the samples of different groups at 4 °C for 10–12 h.

After incubation, membranes were rinsed with washing buffer and incubated with a primary biotin-labeled antibody (provided in the kit) for 2 h and HRP-streptavidin (provided in the kit) for 1.5 h, successively [15].

Afterward, the membranes were washed multiple times with washing buffer and incubated with detection buffer to develop Chemiluminescence. Chemiluminescence was detected using a CCD Camera (INTAS Science Imaging Instruments, Goettingen, Germany). The relative expression of POS (positive control) and NEG (negative control) spots on the membrane was measured densitometrically using ImageJ software [38].

### 2.9. Identification of Candidate Molecules for Signaling Pathways Using the ChEA3 Tool and the KEGG Database

As Keenan et al. described [39], the ChEA3 tool was used to localize transcription factors associated with the targets screened by the angiogenesis array assay. Subsequently, the transcription factors of interest were entered into the Kyoto Encyclopedia of Genes and Genomes (KEGG) database using DAVID tools [40], which in turn screened for possible involvement in signaling pathways.

### 2.10. Western Blot

Western blot analysis was performed as previously described [41]. In brief, HaCaT cells in each group were washed with ice-cold DPBS and then lysed in the ice-cold radioimmunoprecipitation assay buffer. Then, 25 μg protein aliquots were loaded onto sodium dodecyl sulfate −12.5% polyacrylamide gel for electrophoresis and transferred onto nitrocellulose membranes. After blocking with 5% bovine serum albumin solution, the membranes were incubated with respective primary antibodies at 4 °C overnight and then incubated with the HRP-conjugated secondary antibody for 2 h at room temperature. Immunoblots were developed using the ECL ChemoCam Camera (INTAS Science Imaging Instruments, Goettingen, Germany). Densitometric analysis of band intensities was calculated using ImageJ software. Expression levels of the targets were normalized concerning GAPDH band density.

### 2.11. Statistics

Data are presented as box plots (Min to Max with single data points) of at least three independent experiments (*N* ≥ 3) measured as duplicates or triplicates (*n* = 2 or 3). Details are provided in the figure legends. GraphPad Prism Software V9.0.0 (San Diego, CA, USA) was used for statistical analysis. A *p* < 0.05 was taken as a minimum level of significance.

## 3. Results

### 3.1. 3% CSE Did Not Significanty Affect HaCaT Cells Viability

**Figure 1:**
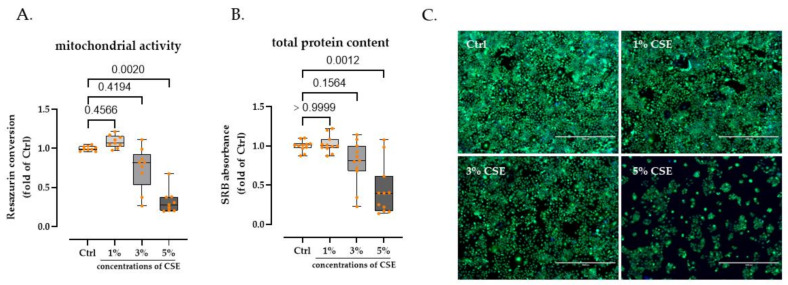
HaCaT cell viability exposed to CSE (0%, 1%, 3%, 5%); 1% and 3% of CSE did not significantly affect the viability, however 5% markedly reduced all three parameters. (**A**) Resazurin conversion was used to quantify cell numbers by mitochondrial activity after 72 h. (**B**) After 72 h, Sulforhodamine B (SRB) staining was used to quantify the total protein content of cells. *N* = 3, *n* = 3. The Kruskal–Wallis H test, followed by Dunn’s post-test, was used to determine statistical significance. (**C**) Representative fluorescent images. Calcein-AM (2 µM, green) was used to visualize live cells.

Concentration was assessed by resazurin conversion assay, SRB staining assay, and calcein-AM/Hoechst staining to detect the effect of CSE on the viability of HaCaT cells; 0% (control group), 1%, 3%, and 5% of CSE exposure were selected. Following 72 h of stimulation, we observed a dose-dependent decrease in mitochondrial activity and total protein content in HaCaT cells after CSE exposure. When the CSE concentration was elevated to 5%, HaCaT cells showed a significant reduction in mitochondrial activity (74.15% decline, *p* = 0.0020) and total protein content (64.73% decline, *p* = 0.0012), respectively. However, 1% and 3% of CSE did not significantly alter the viability of HaCaT cells (Figure 1A,B). Meanwhile, this phenomenon was confirmed and visualized via calcium-AM/Hoechst staining (Figure 1C). 

### 3.2. 3% CSE Significantly Blunted the Angiogenic Potential by HaCaT Cells, and This Was Offset by 100 μA MC Stimulation

**Figure 2:**
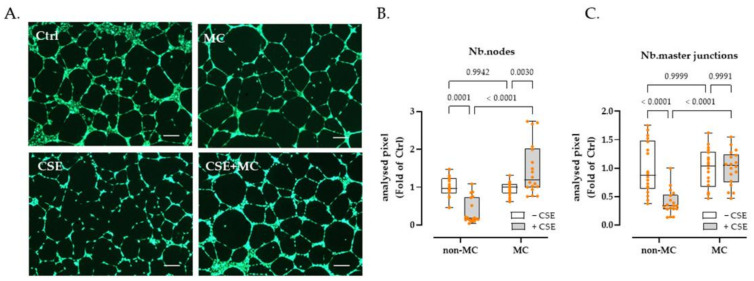
HUVEC cells were used as an indicator to examine changes in the angiogenic capacity of HaCaT cell supernatants after CSE or MC stimulation. (**A**) Representative fluorescent Calcein-AM (2 µM, green) staining showing the effect of HaCaT supernatant collected from different groups on HUVEC tube formation. (**B**,**C**) showed that 3% CSE significantly decreased Nb.nodes (63.82% decline, as x-fold of Ctrl) and Nb.master junction (60.36% decline, as x-fold of Ctrl); 100 µA MC increased the above indices by 396.85% and 154.29% in CSE-injured HaCaT cells; however, no significant improvement of MC was observed in the tube-forming ability of control HaCaT cells. Scales bars indicate 200 µm in length. *N* = 6, *n* = 3. Non-parametric two-way ANOVA followed by Tukey’s multiple comparisons was performed to compare the data.

HUVEC cells were used as an indicator to examine the angiogenic ability of culture supernatants from HaCaT cells cultured under different exposure conditions. The results showed that 3% CSE significantly decreased Nb.nodes (63.82% decline, *p* = 0.0001) and the Nb.master junction (60.36% decline, *p* < 0.0001). Interestingly, exposure to 100 μA MC significantly increased the tube-forming capacity of HaCaT cells damaged by 3% CSE. Under the MC intervention, the Nb.nodes and Nb.master junctions of CSE-injured HaCaT cells increased by 396.85% (*p* < 0.0001) and 154.29% (*p* < 0.0001), respectively. It is worth noting that MC did not improve the tube-forming capacity of control HaCaT cells significantly. In other words, exposure to MC offset the harmful effects of CSE in HaCaT cells, however, it had little effect on control cells in terms of their ability to stimulate angiogenesis (Figure 2B,C).

### 3.3. 100 μA MC Prevents the Detrimental Effects of 3% CSE on the Secretion of Pro-Angiogenic Factors by HaCaT Cells

**Figure 3:**
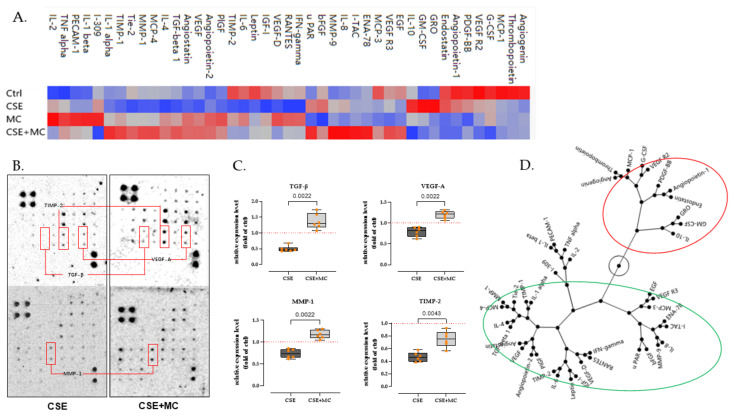
Analysis of angiogenesis-stimulating factors expressed in the supernatant of HaCaT cells after CSE and/or ES exposure. (**A**) The results are presented in a heat map, and the color goes from blue to red with increasing expression values. (**B**) Representative expression of pro-angiogenic factors TGF-β, VEGF-A, MMP-1, and TIPM-2 (shown in red box). (**C**) Data are displayed normalized to the control group’s mean (red dashed line). *N* = 3 (pooled), *n* = 2. A non-parametric Mann–Whitney test was used to compare the data. (**D**) The results of the clustering analysis are presented as a constellation diagram. Group 1 (green circles) indicates targets whose expression was impaired by 3% CSE, and 100 µA MC could prevent this impairment therapeutically. Group 2 (red circles) contains features that differ from Group 1.

To further characterize findings observed in the tube formation assay, we performed the angiogenesis array assay to test the angiogenic-associated factors secreted by HaCaT cells after 3% CSE and/or MC exposure. The results presented as a heat map (Figure 3A) revealed that the secretion of multiple angiogenic-associated factors was markedly disturbed after 3% CSE exposure. Of the 43 examined targets, 37 (86%) exhibited downregulation in the CSE group compared with the control. Some key regulators of angiogenesis, e.g., transforming growth factor-beta (TGF-β, 57.6% decline), vascular endothelial growth factor (VEGF, 18.7% decline), matrix metalloproteinase-1 (MMP-1, 33.0% decline), and tissue inhibitor of metalloproteinases 2 (TIMP-2, 53.9% decline) showed an obvious decrease (Figure 3B,C). Interestingly, MC appeared to mitigate the detrimental effects of CSE and even restored these factors to near baseline levels. The expression levels of the factors shown in the red boxes (Figure 3B,C) were markedly reduced in the presence of 3% CSE, but the expression of these factors (e.g., TGF-β (176.6% increase, *p* = 0.0022), VEGF (49.4% increase, *p* = 0.0022), MMP-1 (57.7% increase, *p* = 0.0022), TIMP-2 (63.0% decline, *p* = 0.0043)) was significantly restored in the presence of 100 μA MC when compared with the 3% CSE group.

### 3.4. Phosphatidyl-Inositol 3-Kinase/Serine-Threonine Kinase (PI3K-Akt), Mitogen-Activated Protein Kinases (MAPK), and Nuclear Factor Kappa-B (NFκB) Signaling Pathway May Be Involved in the Differential Expression of Angiogenic-Related Factors

Based on the clustering results of the constellation plot, we divided the targets present on the array into two groups (Figure 3D). Group 1 (green circles) indicates that the CSE reduced the expression of the targets and that MC can mitigate this damage. Group 2 (red circles), on the other hand, contains targets that do not match or differ from the above characteristics.

Next, we used the ChEA3 tool [39] to retrieve potential transcription factors for the targets in Group 1 and Group 2. Each group’s top 100 transcription factors (Supplemental Material, Supplemental Tables S1 and S2) were extracted as candidates based on Mean Rank (the average ranking obtained by combining multiple databases, from smallest to largest). Given the opposite expression roles of the factors in the two groups, we excluded the overlap between the two groups, and 68 transcription factors were selected (Supplemental Material, Supplemental Table S3) from Group 1.

To further study the potential signaling pathways involved, we performed the KEGG enrichment analysis using DAVID tools [40]. After inputting the screened transcription factors into KEGG, results suggested that PI3K-Akt, MAPK, and NFκB signaling pathways may be activated (Supplemental Material, Supplemental Figure S1).

### 3.5. MC Exposure Intensified PI3K-Akt and MAPK Signaling in CSE-Injured HaCaT Cells

Based on the enrichment analysis results, we investigated the phosphorylation changes of crucial molecules of PI3K-Akt, MAPK, and NFκB signaling pathways after 30 min of CSE and/or MC stimulation using Western blot. In addition, we investigated the phosphorylation of 27HSP, which responded to the level of cellular stress. 

**Figure 4:**
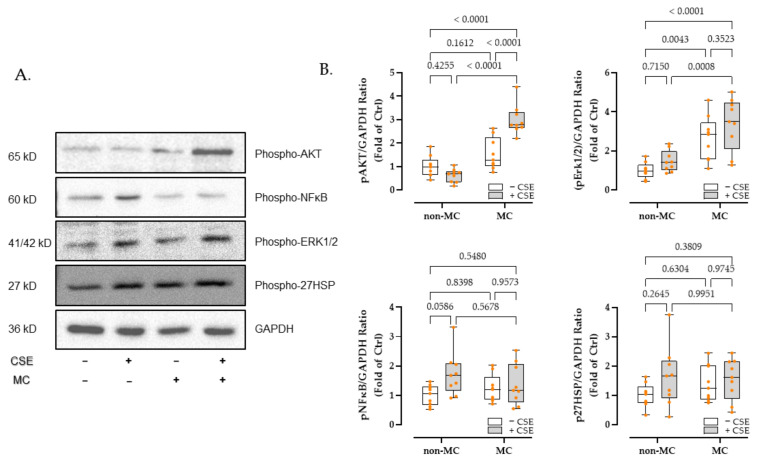
Western blots showed 100 μA MC exposure intensified PI3K-Akt and MAPK signaling in CSE-injured HaCaT Cells. Densitometer readings and related information are provided in Supplementary Table S4. Original Western blot images are provided in Supplementary Figure S2. (**A**) Representative Western blot. (**B**). (**Upper left**) 3% CSE reduced p-Akt by 38.72%, meanwhile, exposure to the 100 μA MC significantly intensified the expression of p-Akt by 299.90% in the presence of CSE. 100 μA MC alone did not significantly enhance the p-Akt expression. (**Upper right**) p-Erk 1/2 100 μA exposure significantly upregulated the p-Erk 1/2 both in the presence or absence of 3% CSE. (**Lower**) no significant changes in p-NFκB and p-27HSP were observed. Quantification of densitometries of the band (as x-fold of Ctrl) was performed via ImageJ Software. GAPDH expressions were used as loading controls. *N* = 3, *n* = 3. Non-parametric two-way ANOVA followed by Tukey’s multiple comparison test was performed to compare the data.

As expected, after 30 min of stimulation, the presence of 3% CSE remarkably reduced (38.72%) the expression of phosphorylated Akt (p-Akt). Exposure to the 100 μA MC significantly intensified the expression of p-Akt by 299.90% (*p <* 0.0001) in the presence of 3% CSE. Interestingly, 100 μA MC alone did not significantly enhance the p-Akt expression (Figure 4B).

In line with this, the signal of phosphorylated Erk 1/2 (p-Erk 1/2) shows an upward trend in all experimental groups when compared to the control group. Furthermore, 100 μA exposure significantly upregulated the phosphorylation of Erk 1/2 by between 260% and 340%, both in the presence or absence of 3% CSE (Figure 4B).

However, no significant changes in critical molecules of inflammation (p-NFκB) and stress-related signaling pathways (p-27HSP) were observed (Figure 4B).

### 3.6. Inhibition of PI3K-Akt Signaling Deprived MC of Its Therapeutic Effect on the Tube Formation Function of CSE-Injured HaCaT Cells

**Figure 5:**
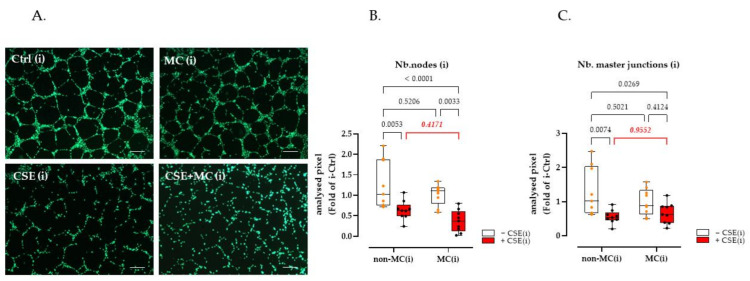
Inhibition of Akt phosphorylation inhibited the therapeutic effect of 100μA MC to enhance CSE-injured angiogenesis. (**A**) Effect on HUVEC tube formation with fluorescent calcein-AM (2 µM, green) staining of formed tubes, scales bars indicate 200 µm length, “i” indicates that the supernatant used for the experiment was collected from SC394003 (inhibitor) pretreated HaCaT cells. (**B**,**C**) The number of nodes and number of master junctions (as x-fold of Ctrl (i)). *N* = 3, *n* = 3. Non-parametric two-way ANOVA followed by Tukey’s multiple comparison test was performed to compare the data.

Since the phosphorylation alteration trend of Akt was similar to that of the tube formation assay and angiogenesis array assay, we performed inhibitor experiments to investigate the possibility of involvement of the PI3K-Akt signaling pathway in angiogenesis. The result demonstrated that the inhibition of Akt signaling by the chemical inhibitor SC394003 (10 μM, Santa Cruz, Dallas, TX, USA) significantly reduced both the Nb.nodes and Nb.master junction in the MC plus CSE group; in other words, after the inhibition of Akt, the therapeutic effect of MC on CSE-injured angiogenesis injury (see Figure 2) was lost (Figure 5). However, no significant inhibitory effect was observed in other groups (Figure 5B,C) and non-inhibitor groups (Figure S3).

## 4. Discussion

Tobacco use is linked to over 60 diseases [3], and it has long been common knowledge that tobacco use has a wide range of adverse effects on the circulatory system [42]. The microcirculation of the skin is also affected by cigarette smoke exposure [43,44]. Cyril et al. demonstrated that the vasodilatory response of the skin microvasculature is significantly impaired in people who have smoked for years [45], and an in vivo study by Sophie-Élise et al. also showed that angiogenesis was impaired considerably in mice exposed to cigarette smoke following surgically induced hindlimb ischemia. Here, we observed a significant decrease in cellular activity when keratinocytes were exposed to 5% CSE, which is equivalent to smoking roughly ten cigarettes (0.5 packs) a day [46]; furthermore, we found that 3% of CSE had no significant impact on keratinocyte viability but significantly impaired its tube-forming capacity. Many clinical studies [47,48] have revealed the necessity of smoking cessation after surgery, especially with regards to microsurgery and wound healing. The findings presented here provide support this clinical conclusion at the cellular level.

Damage to the epithelium results in the instantaneous generation of endogenous electric fields (created by the transepithelial potential difference) at the site of injury, a phenomenon discovered in humans more than 150 years ago in skin wounds [14]. It is gradually being recognized that this electrical signal is the key to guiding the whole process of wound healing [49,50,51]. ES has been used in clinical settings for over half a decade to support wound healing [22]. The present study utilized MC (100 μA) to investigate its therapeutic effect on the angiogenesis of CSE-treated keratinocytes, and the results showed that exposure for 72 h significantly restored the angiogenic potential of HaCaT cells damaged by 3% CSE. Our findings are consistent with an in vivo experimental study by Michaud et al., who showed significant angiogenic impairment in mice exposed to cigarette smoke following surgically induced hindlimb ischemia [52], and the reason may be related to the inhibition of angiogenesis by cigarette smoke, where the expression of hypoxia-inducible factor-1α (HIF-1α) and vascular endothelial growth factor (VEGF) is reduced under hypoxic conditions. In addition, an in vivo study completed by Souza et al. also confirmed that elevated angiogenesis could be observed in a rat skin model pretreated with nicotine following ES application [53]. Furthermore, for the effect of ES on angiogenesis-related cells, Bai et al. demonstrated that the elongation, orientation, and migration of vascular endothelial cells can be clearly affected by DC electric fields [54], and that endothelial cells from angiogenic microvessels respond more strongly to electrical stimulation compared to non-angiogenic macrovessels [55], which supports the potential of ES to promote healing by accelerating angiogenesis.

It is worth noting that angiogenesis can be a double-edged sword, as it is essential in promoting wound healing and can also increase the risk of tumorigenesis. In our study, the pro-angiogenic effect of MC was significantly increased only in cigarette-damaged keratinocytes, while no significant changes were triggered in normal cells, suggesting the safety of MC when applied to normal tissues. 

During skin or tissue healing, keratinocytes produce various cytokines, growth factors, and chemokines involved in wound healing, especially angiogenesis [56]. It has been reported that cigarette inhalation impairs angiogenesis by disrupting the expression of angiogenic-related factors during early healing progress [9], which may even cause delayed ulcer healing [57]. In our study, we observed that 72 h of 3% CSE stimulation significantly downregulated the secretion of a series of factors contributing to angiogenesis in HaCaT cells; moreover, exposure to 100 μA of MC therapeutically restored the impairment of expression of these factors, and these findings are consistent with the studies mentioned above. In particular, we showed significant restoration of the expression of certain factors, such as TGF-β, VEGF, MMP-1, and TIMP-2.

TGF-β is involved in a multitude of wound healing mechanisms. In the early stages of injury, it is released by keratinocytes, platelets, and macrophages and plays a role in regulating inflammation, stimulating angiogenesis, and regeneration of the extracellular matrix (ECM) [58,59]. Our study confirms that MC can protectively upregulate cigarette-mediated impairment of TGF- β secretion, which we speculate may be related to primary cilia. Primary cilia are considered the “mechanosensory tentacles” of almost all mammalian cells, a specialized cellular sensory organ that transmits information about the environment to the cell. A recent study in our laboratory by Aspera-Werz et al. has shown that cigarette smoke interferes with TGF-β signaling in human mesenchymal stem cells by impairing the structural integrity of primary cilia, thereby inducing the risk of metabolic bone disease [46]. Notably, our colleagues Chen et al. showed that exposure to electromagnetic fields protects the integrity of primary cilia structures in osteoprogenitors injured by cigarette smoke [60], reflecting our results.

Regarding angiogenesis, VEGF is undoubtedly the most studied and crucial endothelial stimulator [10]. VEGF is expressed mainly in endothelial cells, macrophages, mesenchymal stem cells, and keratinocytes [61,62], which explains their presence in our model. During wound healing, VEGF plays a critical role in stimulating angiogenesis. Studies demonstrated that healing in non-diabetic ischemic wounds is improved by VEGF administration [63]; blocking VEGF with neutralizing antibodies impedes healing [64]. One study showed that mice exposed to cigarette smoke show lower levels of VEGF in fracture callus and higher rates of non-healing fractures [9]. In our study, 3% CSE impaired VEGF secretion from HaCaT cells, which may have contributed to the observations of the tube formation assay. Meanwhile, we observed that 100 μA MC significantly increased the expression of VEGF damaged by CSE, and this is consistent with the study of Souza et al. In her research, she confirmed that ES improved skin survival after skin grafting in rats, showing a lower percentage of tissue shrinkage, less inflammatory infiltration, and more VEGF, when compared to the nicotine pre-stimulation group [53]. 

Additionally, in 3% CSE-treated HaCaT cell supernatants, we observed a decrease in MMPs and TIMPs, suggesting that cigarettes may impair the matrix remodeling capacity and, thus, potentially affect angiogenesis and healing. MMPs are protein hydrolases for extracellular matrix (ECM) degradation, which are involved in leveling the growth pathway of neovascularization and increasing the bioavailability of growth factors [65]. TIMPs are natural inhibitors of MMPs, and the interaction between MMPs and TIMPs determines the deposition and degradation of the ECM, which is essential for neovascularization during healing [66]. It has been suggested that disturbed expression of TIMPs and MMPs may delay wound healing [67], and this phenomenon can be clearly observed in the serum of smokers [68]. In our study, we observed that 100 μA MC corrected the expression of MMPs and TIMPs that were downregulated by 3% CSE, and CSE-injured HaCaT cells showed a higher magnitude of upregulation of MMPs compared to TIMPs after MC exposure, suggesting a therapeutic role of MC in enhancing matrix remodeling potential, which is beneficial for angiogenesis. 

Multiple cellular and organ functions are mediated by PI3K/Akt signaling, including cell survival, protein synthesis, etc. [69]. In our study, we observed a downregulation of Akt phosphorylation due to 3% CSE exposure, suggesting that cigarettes impair the angiogenic function of HaCaT cells through phosphorylation of Akt. This is in line with Kim et al., who demonstrated in a rat model that 4% CSE significantly downregulated Akt phosphorylation in fibroblasts, leading to impaired apoptosis, senescence, and repair function [70]. At the same time, there is clear evidence that the PI3K-Akt signaling pathway is involved in the electrical signaling response. A study from Zimolag et al. demonstrated that disruption of the PI3K-Akt signaling pathway rapidly affected the migration of MSCs towards the cathode [71], and Wang et al. also demonstrated that ES promoted the proliferation of HUVECs in a diabetic model by activating the PI3K-Akt signaling pathway, while simultaneously upregulating their VEGF expression [72].

Through inhibitor experiments, we have clarified the crucial role of MC-mediated PI3K-Akt alterations in repairing cigarette-induced keratinocyte tube-forming impairment. At the same time, the synergistic role of MAPK in this process cannot be ignored. In this study, we observed the upregulation of Erk1/2 in the MC group, regardless of the presence or absence of CSE; this is consistent with our previous study [15]. In addition, Li et al. demonstrated a significant response of PI3K-Akt and MAPK pathways of epidermal stem cells under electric field stimulation, which synergistically promoted their directional migration [73]. However, phosphorylated NFκB and 27HSP did not show significant alterations across groups compared to controls, suggesting that short periods (72 h) of cigarette stimulation or MC stimulation did not result in a robust cellular inflammatory or stress response.

## 5. Conclusions

Our study shows that 3% CSE (equivalent to smoking six cigarettes per day) negatively affects the tube-forming capacity and the expression of pro-angiogenic factors of HaCaT cells. A continuous 72 h of exposure to 100 μA MC partially reversed the harmful effects of CSE. Further, MC-mediated alterations in the PI3K-Akt signaling pathway were identified as a potential regulatory mechanism. In conclusion, our results suggest that continuous MC exposure for 72 h can support early wound healing by promoting vasculature regeneration of cigarette-injured skin cells. This will provide theoretical and mechanistic support for ES treatment strategies for clinical wound healing dilemmas in smokers.

## Data Availability

The data that support the findings of this study are available upon reasonable request from the corresponding author.

## Supplementary Materials

The following supporting information can be downloaded at: https://www.mdpi.com/article/10.3390/bioengineering9090445/s1, Figure S1: KEGG enrichment analysis suggested that PI3K-AKT, MAPK, and NF-κB signaling pathways may be activated; Figure S2: Representative Western blot and the corresponding plain images for phospho-AKT, phospho-NFκB, phospho-Erk1/2, and phospho-HSP27 in HaCaT cells 30min after 100 μA MC exposure; Figure S3: The addition of PI3K-AKT signaling pathway inhibitor SC394003 significantly reduced the levels of tube-forming indicators in the MC+CSE group compared with the non-inhibitor groups, whereas in the other groups, the inhibitory effect of this inhibitor on tube-forming indicators was not significant. Table S1: Top 100 transcription factors in Group 1; Table S2: Top 100 transcription factors in Group 2; Table S3: The 68 transcription factors in Group 1 after excluding the overlap with Group 2; Table S4: Densitometry Readings/intensity Ratio of western blot.

## Abbreviations

CS	cigarette smoking
CSE	cigarette smoke extract
ECL	enhanced chemiluminescence
ES	electrical stimulation
FBS	fetal bovine serum
HaCaT	human keratinocyte cell line
MAPK	mitogen-activated protein kinases
MC	microcurrent
MMP-1	matrix metalloproteinase-1
NFκB	nuclear factor kappa-B
PI3K-Akt	phosphatidyl-inositol 3-kinase/serine-threonine kinase
SRB	sulforhodamine B
TEP	transepithelial potential difference
TGF-β	transforming growth factor beta
TIMP-2	tissue inhibitor of metalloproteinases 2
