# Supplementary material for: Microcurrent Reverses Cigarette Smoke-Induced Angiogenesis Impairment in Human Keratinocytes In Vitro
Source: Bioengineering (Basel). 2022 Sep 6;9(9):445. doi: 10.3390/bioengineering9090445 (PMC9495747; doi:10.3390/bioengineering9090445)
Supplement: Supplementary file 1 [file bioengineering-09-00445-s001.zip › bioengineering-1880915-supplementary.pdf]

bioengineering

MDPI

Supplementary marital: Microcurrent Reverses Cigarette Smoke-Induced Angiogenesis Impairment in Human Keratinocytes In Vitro

Table S1. Top 100 transcription factors in Group 1.

Rank	TF	Score
1	SNAI1	11.33
2	BCL6B	17.33
3	CSRNP1	37
4	FOXD1	37.5
5	NR1H3	38.8
6	CEBPB	46.33
7	RFX8	47.5
8	PPARG	48.2
9	NFKB2	50.5
10	ARID5A	52
11	RUNX3	57.2
12	RELB	58.5
13	TCF21	61.5
14	TBX21	67.5
15	FOXC2	67.67
16	FOSL1	71.6
17	SNAI2	72.5
18	FOXS1	74.67
19	SOX17	75.5
20	PPARD	78.75
21	BATF3	91
22	ZNF469	95
23	EPAS1	95.5
24	PBX4	96.67
25	FOXP3	98.75
26	EGR2	99
27	GATA2	100.5
28	ZNF267	101.3
29	MEOX1	101.3
30	ETV3L	102.7
31	GLIS2	104.7
32	PRRX1	105.7
33	JUNB	108.8
34	PRRX2	110
35	SOX7	110
36	NR4A3	111
37	ETS1	112.3
38	C11ORF95	113
39	MEOX2	115.3

40	SMAD3	115.6
41	TFE3	116.7
42	MTF1	124.5
43	CEBPA	125.4
44	ZBED3	126
45	FOSL2	128
46	KLF2	129.3
47	NFE2L2	129.4
48	ERG	131.6
49	EOMES	136.2
50	IRF1	136.7
51	TAL1	140
52	SOX18	140.3
53	CREB3L2	140.7
54	AEBP1	141.5
55	IKZF1	143.3
56	STAT5A	145.3
57	HLX	148
58	GTF2B	149.3
59	ZBED2	151.3
60	MAFB	151.5
61	ZNF792	152
62	ELK3	154
63	STAT4	156.5
64	TEAD1	159.3
65	GLMP	162.5
66	ZNF366	165.3
67	HOXD8	165.7
68	SPI1	165.8
69	TCF7	169
70	HOXD4	169.7
71	ZNF80	170
72	KLF4	171
73	HHEX	171.5
74	HMGA2	174.3
75	GFI1	176.3
76	ZNF587B	178
77	RARA	180.4
78	NFKB1	182
79	IRF7	183
80	NFIL3	183.3
81	NR2F2	184.4
82	POU2F2	184.6
83	MYC	186
84	RELA	186.2
85	FOXF1	191.3
86	ZBTB14	192

87	LYL1	192.8
88	HOXC6	193.3
89	KDM2A	193.5
90	BATF	195.2
91	IRF4	196
92	STAT1	199
93	RBPJL	200
94	RUNX1	201
95	REL	202
96	PRDM1	203.4
97	TFAP2A	204.8
98	ZNF385A	205
99	FOXO1	205.6
100	NFATC4	206

Table S2. Top 100 transcription factors in Group 2.

Rank	TF	Score
1	SOX18	9.333
2	BCL6B	11.67
3	GATA2	36.67
4	SOX17	37.25
5	SOX7	41.33
6	NR1H4	51.67
7	ERG	53.8
8	ELK3	60.75
9	SLC2A4RG	64.67
10	AEBP1	72.5
11	EPAS1	74.5
12	GLIS2	77.67
13	HEYL	81
14	ARID3C	86.5
15	CREB3L3	89.67
16	HNF1B	90
17	GSC	96
18	HHEX	98.75
19	NKX23	101
20	HEY2	106.3
21	SHOX2	107
22	HNF4A	110.7
23	SOX13	114.5
24	PAX8	116.8
25	ZNF467	125
26	BORCS8MEF2B	126
27	CEBPE	127.7
28	NR2F2	134.2
29	NFE2	134.4
30	SMAD3	135.2

31	HAND1	139
32	CENPX	141
33	ZNF641	142.3
34	LBX2	145
35	FOXA3	146.7
36	BCL6	153.8
37	NFE4	154
38	ATF3	158.7
39	SPI1	161.3
40	SNAI1	162.3
41	LHX1	163
42	CREB5	168.7
43	SIX5	173.2
44	DUX4	175
45	NR2F6	176.8
46	NR5A2	179.5
47	HOXC4	180.3
48	HNF1A	180.3
49	USF1	180.4
50	CEBPD	182
51	EBF3	182
52	LHX6	182.3
53	MEIS1	183.2
54	ETV3L	184.3
55	RARA	185.2
56	NR4A3	193.3
57	MLXIPL	195
58	HES1	196
59	SP110	200.3
60	HOXA6	207
61	ATF5	209
62	GCM1	210
63	TCF15	210
64	TEAD2	210.5
65	HOXC6	212.3
66	HLX	214.7
67	TBX2	215.3
68	TEAD4	218.7
69	EGR2	218.7
70	IRF4	218.8
71	ANHX	220.5
72	NR1I2	221.3
73	ZNF366	225.3
74	CEBPB	226.2
75	STAT3	226.2
76	TFAP2A	227.3
77	KDM5B	227.4

78	IRF1	230.2
79	NFE2L2	234
80	ZIC3	235.3
81	TET2	236
82	FOXC1	236.3
83	HOXB4	236.8
84	KLF4	237.6
85	FOSL2	240.4
86	ELF1	242
87	ZNF792	243.7
88	HOXB3	244.3
89	LHX9	244.7
90	TBX21	250.5
91	SRY	250.8
92	LYL1	252.6
93	GFI1B	253.6
94	ZNF764	256.7
95	ZNF319	258.3
96	ZFHX4	258.3
97	HOXA9	261.5
98	GATA4	262.6
99	GATA3	263.5
100	FLI1	266

Table S3. The 68 transcription factors in Group 1 after excluding the overlap with Group 2.

Number	TF
1	CSRNP1
2	FOXD1
3	NR1H3
4	RFX8
5	PPARG
6	NFKB2
7	ARID5A
8	RUNX3
9	RELB
10	TCF21
11	FOXC2
12	FOSL1
13	SNAI2
14	FOXS1
15	PPARD
16	BATF3
17	ZNF469
18	PBX4
19	FOXP3
20	ZNF267
21	MEOX1
22	PRRX1
23	JUNB
24	PRRX2

25	ETS1
26	C11ORF95
27	MEOX2
28	TFE3
29	MTF1
30	CEBPA
31	ZBED3
32	KLF2
33	EOMES
34	TAL1
35	CREB3L2
36	IKZF1
37	STAT5A
38	GTF2B
39	ZBED2
40	MAFB
41	STAT4
42	TEAD1
43	GLMP
44	HOXD8
45	TCF7
46	HOXD4
47	ZNF80
48	HMGA2
49	GFI1
50	ZNF587B
51	NFKB1
52	IRF7
53	NFIL3
54	POU2F2
55	MYC
56	RELA
57	FOXF1
58	ZBTB14
59	KDM2A
60	BATF
61	STAT1
62	RBPJL
63	RUNX1
64	REL
65	PRDM1
66	ZNF385A
67	FOXO1
68	NFATC4

Table S4. Densitometry Readings/intensity Ratio of western blot.

	**pAkt / GAPDH**	**pErk1/2 / GAPDH**	**pNFkB / GAPDH**	**p27HSP / GAPDH**
Ctrl	1.000	1.00000	1.00000	1.00000
CSE	0.6123	1.470	1.725	1.633
MC	1.538	2.640	1.228	1.408
MC+CSE	2.990	3.384	1.367	1.551

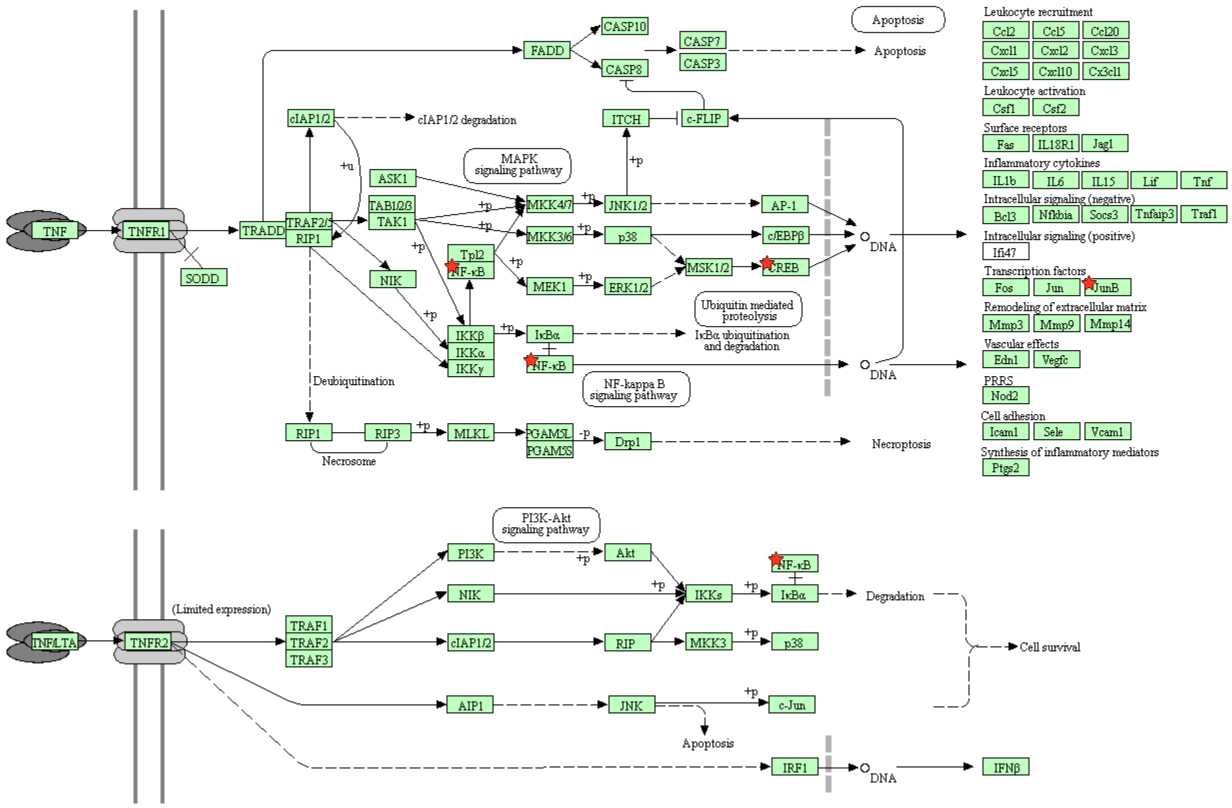

Figure S1. KEGG enrichment analysis suggested that PI3K-AKT, MAPK, and NF-κB signaling pathways may be activated. Stars indicate the transcription factors were enriched.

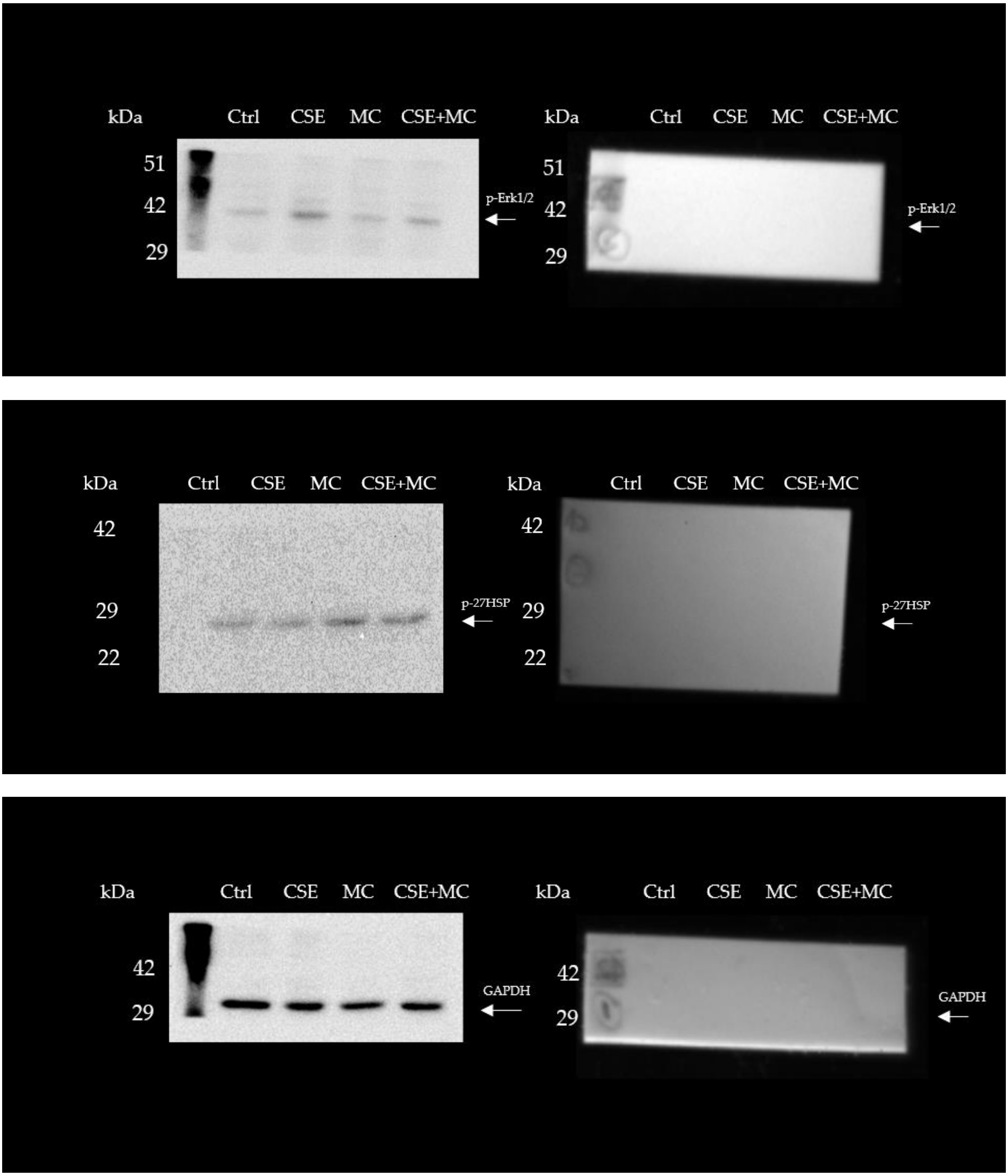

Figure S2. Representative Western blot (left) and the corresponding plain images(right) for phospho-AKT, phospho-NFκB, phospho-Erk1/2, and phospho-HSP27 in HaCaT cells 30 min after 100 μA MC exposure. The expression of GAPDH was used as a loading control.

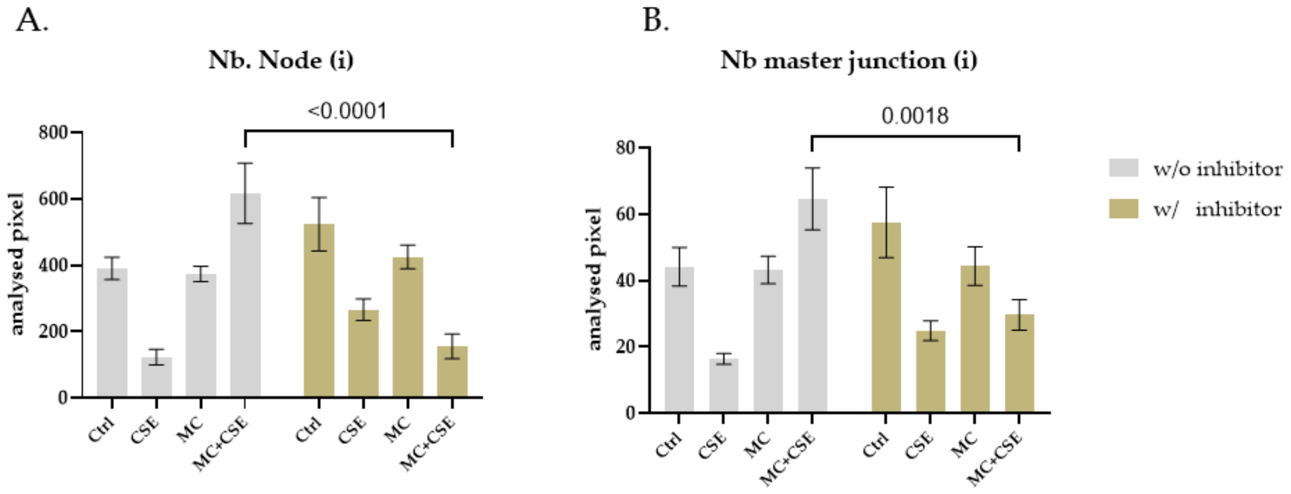

Figure S3. The addition of PI3K-AKT signaling pathway inhibitor SC394003 significantly reduced the levels of tube-forming indicators (A) Nb.Node and (B) Nb.Node in the MC+CSE group compared with the non-inhibitor groups, whereas in the other groups, the inhibitory effect of this inhibitor on tube-forming indicators was not significant. N = 3, n = 3. Data were compared by non-parametric two-way ANOVA followed by Tukey's multiple comparison tests.
